# Novel microRNA discovery using small RNA sequencing in post-mortem human brain

**DOI:** 10.1186/s12864-016-3114-3

**Published:** 2016-10-04

**Authors:** Christian Wake, Adam Labadorf, Alexandra Dumitriu, Andrew G. Hoss, Joli Bregu, Kenneth H. Albrecht, Anita L. DeStefano, Richard H. Myers

**Affiliations:** 1 Department of Neurology, Boston University School of Medicine, Boston, USA; 2 Bioinformatics Program, Boston University, Boston, USA; 3 Graduate Program in Genetics and Genomics, Boston University School of Medicine, Boston, USA; 4 Genome Science Institute, Boston University School of Medicine, Boston, USA; 5 Section of Biomedical Genetics, Department of Medicine, Boston University School of Medicine, Boston, USA; 6 Department of Biostatistics, Boston University School of Public Health, Boston, USA

**Keywords:** MicroRNA, miRNA sequencing, Novel miRNA discovery, Prefrontal cortex, Neurodegenerative disease

## Abstract

**Background:**

MicroRNAs (miRNAs) are short, non-coding RNAs that regulate gene expression mainly through translational repression of target mRNA molecules. More than 2700 human miRNAs have been identified and some are known to be associated with disease phenotypes and to display tissue-specific patterns of expression.

**Methods:**

We used high-throughput small RNA sequencing to discover novel miRNAs in 93 human post-mortem prefrontal cortex samples from individuals with Huntington’s disease (*n* = 28) or Parkinson’s disease (*n* = 29) and controls without neurological impairment (*n* = 36). A custom miRNA identification analysis pipeline was built, which utilizes miRDeep* miRNA identification and result filtering based on false positive rate estimates.

**Results:**

Ninety-nine novel miRNA candidates with a false positive rate of less than 5 % were identified. Thirty-four of the candidate miRNAs show sequence similarity with known mature miRNA sequences and may be novel members of known miRNA families, while the remaining 65 may constitute previously undiscovered families of miRNAs. Nineteen of the 99 candidate miRNAs were replicated using independent, publicly-available human brain RNA-sequencing samples, and seven were experimentally validated using qPCR.

**Conclusions:**

We have used small RNA sequencing to identify 99 putative novel miRNAs that are present in human brain samples.

**Electronic supplementary material:**

The online version of this article (doi:10.1186/s12864-016-3114-3) contains supplementary material, which is available to authorized users.

## Background

MicroRNAs (miRNAs) are a class of small, noncoding regulatory RNAs. In their mature form, miRNAs are single-stranded, 19–23 nucleotides in length, most often generated from hairpin precursors transcribed from intergenic, intronic, or exonic regions of the genome. Mature miRNAs act as post-transcriptional regulators of gene expression through sequence-specific targeted binding of mRNA. The most common mechanism of regulation by miRNAs is translational repression of targeted mRNA. A single miRNA may target multiple mRNAs and a single mRNA may be targeted by multiple miRNAs [1, 2].

The importance of miRNA regulation of mRNAs and its effect on organism phenotype is not yet fully understood, but it is clear that it is a widespread phenomenon [3]. miRNAs are found in plants and animals and function in a variety of biological processes including cell growth, proliferation and apoptosis [4]. It has been estimated that more than half of all mammalian protein-coding mRNAs experience some degree of miRNA regulation [5]. Considering this, it is unsurprising that the deregulation of miRNAs has been associated with many diseases, including neurodegenerative diseases such as Huntington’s disease and Parkinson’s disease [6, 7].

miRNAs can be species-specific and many display tissue-specific patterns of expression [3, 8]. More than 2700 known human mature miRNAs are documented in miRBase v20 [9], a number that has increased rapidly in the past, due largely to the increasing availability and quality of next-generation sequencing yielding larger numbers of RNA-seq data sets from a wider variety of tissue types [10]. Nonetheless, few human brain samples have been mined for the discovery of novel miRNAs, relative to more available tissue types. This yields the possibility that there are brain-specific and possibly human-specific miRNAs that have yet to be discovered.

The goal of this study was to identify novel, potentially brain-specific miRNA species using miRNA sequencing from 93 human prefrontal cortex tissue samples, comprising persons with Huntington’s disease (HD) or Parkinson’s disease (PD) and neurologically healthy controls. Novel miRNAs were identified using the miRDeep* procedure [11] and final results were annotated for sequence similarity to known miRNAs, proximity to genes, and potential expression differences between disease and control samples. A portion of the putative novel miRNAs were experimentally validated from a set of independent samples.

## Methods

**Fig. 1:**
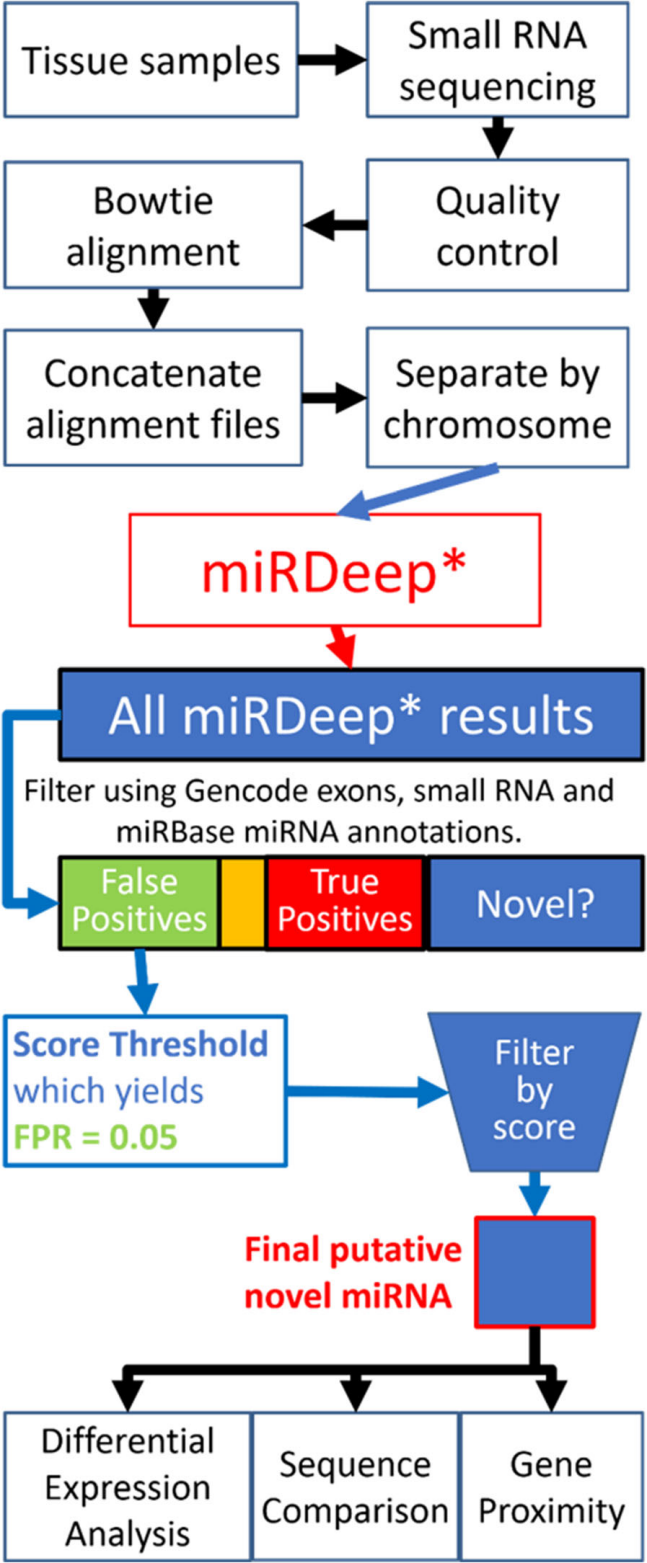
Flowchart depiction of the novel miRNA discovery pipeline. This flowchart shows our novel miRNA discovery pipeline beginning with the small RNA sequencing of tissue samples, quality control (cutadapt v. 1.2.1, FASTX-Toolkit v. 0.0.14) and alignment of sequencing reads in fastq file format, and preparation of alignment (bam) files for miRDeep*. The miRNA predictions made by miRDeep* were classified as false positives (gencode v19 exons/sRNA), true positives (miRBase v20 miRNA), or potential novel miRNAs, which were filtered further. In the flowchart, the yellow box represents miRDeep* results that occurred on both exon and miRNA annotations, which were excluded from the analysis. The false positives were used to determine a miRDeep* score threshold with a false positive rate close to 0.05, which was used to filter the miRDeep* results that are neither false positives nor true positives. The result of this filtering step was a final set of putative novel miRNAs. This set was annotated with differential expression analysis (HD/C and PD/C), alignment (SSEARCH) of mature miRNA sequences to known human mature miRNA sequences in miRBase v20, and proximity to human genes

Sample Information is provided in Additional file 1 and summarized in Table 1. The pipeline methods are depicted as a flowchart in Fig. 1.

**Table 1:** Sample information

Covariates	Huntington’s disease (*N* = 28)	Parkinson’s disease (*N* = 29)	Controls (*N* = 36)
Mean (range) Age at death [years]	60.1 (40–86)	77.6 (64–95)	68.6 (40–97)
Mean (range) PMI^a^ [hours]	16.7 (4–37)	11.1 (1–31)	14.4 (2–28)
Mean (range) RIN^b^	7.3 (6.0–9.2)	7.3 (5.5–8.5)	7.7 (6.3–8.7)
% male	93 %	100 %	92 %

^a^Postmortem interval

^b^RNA Integrity Number

### Sample preparation and sequencing

Frozen brain tissue from the prefrontal cortex Brodmann Area 9 (BA9) was available from three brain banks: the National Brain and Tissue Resource for Parkinson’s Disease and Related Disorders at Banner Sun Health Research Institute, Sun City, Arizona, the Harvard Brain Tissue Resource Center McLean Hospital, Belmont, Massachusetts, and the Human Brain and Spinal Fluid Resource Center VA, West Los Angeles Healthcare Center, California.

Isolation and purification of total RNA was done using QIAzol Lysis Reagent and miRNeasy MinElute Cleanup columns, respectively. RNA quality was assessed either by RNA Quality number from Agilent’s BioAnalyzer 2100 and RNA 6000 Nano Kits, or by RNA Quality Number from Agilent 2200 TapeStation and Agilent DNA ScreenTape assay. Sequencing libraries were constructed using 1 μg of RNA per sample and Illumina’s TruSeq Small RNA Sample Prep Kit with the manufacturer’s protocol. Illumina’s HiSeq 2000 system was used for 1×51 nucleotides, single-end sequencing, conducted at Tufts University (http://tucf-genomics.tufts.edu/) or Michigan State sequencing core facility (http://rtsf.natsci.msu.edu/genomics/). Batch 1 and batch 2 used eight samples per lane with equal numbers of HD and control samples per lane. In batch 3, all samples were given unique bar-codes and distributed across all lanes, with the number of lanes and samples allocated to reach the target read depth of 16,000,000 reads per sample. Additional file 2 contains per sample raw read depth and quality control information.

### Sequencing analysis

Adaptor sequences were trimmed from the reads using cutadapt v. 1.2.1 [12], discarding reads with length less than 15 nucleotides and longer than 27 nucleotides. Sequencing reads whose length was greater than 80 % bases with Phred quality score less than 20 were removed using the fastq_quality_filter command line function from FASTX-Toolkit (version 0.0.14) [13]. Alignment of miRNA sequencing reads to the human reference genome build hg19 was performed using Bowtie v. 1.1.1 [14], allowing no mismatches (*v* = 0) and up to 200 instances of multi-mapping (*m* = 200). More stringent read length filtering was done by miRDeep* before identification of novel miRNAs, discarding reads with length less than 18 nucleotides and greater than 23 nucleotides, miRDeep* default parameters. Differential expression analyses used the less stringent length filtered reads. Additional file 2 contains per sample quality control information including the number of reads used in miRNA discovery and in differential expression analyses.

### Identification of novel miRNAs

The identification of miRNAs was performed using the tool miRDeep* v. 35 [11]. miRDeep* determines the location of putative miRNAs based on aligned small RNA sequencing reads, and scores each prediction with a measure of the likelihood that the miRDeep* result is a true miRNA. The miRDeep* score takes into account artifacts of miRNA precursor processing by Dicer and Drosha, including the positions, lengths and relative frequencies of reads that align to the three precursor products (mature miRNA, star sequence and hairpin loop), and the presence of a short 3’ overhang on the mature sequence. In addition, the score is increased by larger read depth, the evolutionary conservation of the 5’ end of the mature sequence, and the stability of the predicted miRNA hairpin secondary structure.

The alignment files from all 93 samples were concatenated, divided by chromosome into 25 separate alignment files, and sorted. miRDeep* was run on each chromosome separately. The 99 final miRDeep* results were those that were both novel and passed the miRDeep* score threshold, which was determined using estimations of the false positive rate (FPR) and true positive rate (TPR). To make these estimations, the miRDeep* predictions for each chromosome were combined and divided into three categories based on hairpin mapping location: 1) known exons and small RNAs other than miRNAs, 2) known hairpin miRNAs, and 3) results that map to the remainder of the genome. This categorization was done using bedtools v2.22.1 [15] with annotation files for exons and small RNAs from gencode v19 [16] and miRNAs from miRBase v20 [9]. Instances where the exon and miRNA annotations overlapped were excluded, because we could not be certain whether there is a true miRNA within an exon or one of the annotations is inaccurate.

For every integer score value between the minimum and maximum scores of miRDeep* miRNA predictions, the FPR and TPR were estimated using the categorizations of feature overlap described above. The FPR was calculated as the number of miRDeep* predictions with score above the threshold overlapping exon or small RNA annotations, divided by the total number of miRDeep* predictions overlapping exon or small RNA annotations. Similarly, the TPR was calculated as the number of miRDeep* predictions overlapping known miRNAs with score above the threshold, divided by the total number of miRDeep* predictions overlapping known miRNAs. We chose the integer score threshold that yielded a FPR nearest 0.05. In order to get a more precise value than the integer score, we repeated this procedure with non-integer thresholds +/- 1 at 0.01 increments of the integer threshold.

### Sequence similarity with known miRNAs

The miRDeep* novel mature sequences were aligned to mature miRNA sequences in miRBase v20 using the SSEARCH alignment algorithm from the FASTA program package [17], filtering results for e-value < 0.05 and nucleotide overlap > = 15. For the novel miRNA and miRBase miRNA pairs with high-quality alignments of mature sequences, the hairpin sequences were aligned using SSEARCH as well. The quality of alignment on the miRNA seed sequence (positions 2–7 from the 5’ end of the mature sequence) was recorded with the number of mismatches or gaps within each seed sequence. Pairwise alignments of miRDeep* novel sequences were performed in the same way as alignments to miRBase entries.

### Differential expression analysis of Huntington’s disease/control and Parkinson’s disease/control

Differential expression analysis was performed with a set of miRNAs including the novel putative miRNAs identified in this study together with known human mature miRNAs from miRBase v20. The results of the novel miRNAs are presented here, and the differential expression of known miRNAs within these data has been previously characterized and published for both HD/control [7] and PD/control [18]. Small RNA sequencing reads were counted using htseq-count from the Python package htseq v. 0.6.1 [19]. miRNAs with 0 read counts in more than half of the samples were disregarded by the differential expression analysis. A variance-stabilizing transformation (VST) was performed using DESeq2 v. 1.6.3 [20], and batch correction was performed using ComBat [21] from the Bioconductor package sva v. 3.12.0 [22] with default options and with all samples. The differential expression analysis was done using linear models implemented using the Bionconductor package LIMMA v. 3.22.7 [23], adjusting for age of death and with FDR-adjusted *p*-values. Bioconductor v. 3.0 and R v. 3.1.1 were used in these analyses. The HD/C (Huntington’s disease and control) analysis was conducted with 36 control and 28 HD brains, and the PD/C (Parkinson’s disease and control) analysis with the same 36 control brains and 29 PD brains. The differential expression analysis is limited by the data’s origination from separate studies, introducing potential biases on gene expression levels, though mitigated however possible.

### Replication of novel microRNAs

Our putative novel miRNAs were replicated using two publicly available small RNA sequencing data sets, obtained from the Gene Expression Omnibus. Santa-Maria et al. (GSE63501) contains 16 human post-mortem Brodmann Area 9 small RNA sequencing samples from individuals with Alzheimer’s disease, tangle-predominant dementia and normal neuropathology. Hebert et al. (GSE46131) contains 20 human post-mortem superior and mid-temporal neocortex grey matter small RNA sequencing samples from individuals with Alzheimer’s disease, dementia with Lewy bodies, hippocampal sclerosis of aging, frontotemporal lobar dementia (FTLD) and normal neuropathology. All 36 samples from these studies were concatenated for our replication analysis. miRNA results from the original and replication analyses were compared by genomic location using the bedtools v2.22.1 intersect function.

### Experimental validation of novel microRNAs

Seven putative novel miRNAs were selected for orthogonal validation using the Exiqon miRCURY LNA Universal RT microRNA PCR system. RNA for these analyses was purified and extracted, as described above, from a set of twelve independent brains. The putative novel miRNAs selected for qRT-PCR analysis represent a wide range of mean expression values (5-5708 mean reads). We prioritized novel miRNAs with significant differential expression. Exiqon custom microRNA LNA PCR primers were designed using the miRNA primer design algorithm available online at exiqon.com. Total RNA was DNased using the TURBO DNA-free Kit following the manufacturer’s instructions (Ambion/Thermo Fisher Scientific). cDNA was generated from 100 ng of RNA using the Universal cDNA synthesis kit II and RNA Spike-in kit following the manufacturer’s instructions (Exiqon). For each sample, a cDNA reaction was performed omitting reverse transcriptase (-RT reaction). qPCR was performed using the ExiLENT SYBR Green Master mix kit following the manufacturer’s instructions (Exiqon) and using either a 1:80 or 1:20 dilution of the cDNA reaction as template. Two + RT reactions and one -RT reaction were performed for each sample and primer set. qPCR was done on a StepOnePlus Real-Time PCR System (ABI/Thermo Fisher Scientific) using the cycling and analysis parameters recommended by Exiqon (Instruction manual v6.1).

## Results

miRDeep* analysis of sequence alignment data from 93 pooled prefrontal cortex samples revealed 8891 miRNAs. Out of these, 3641 remained after filtering for known miRNAs from miRBase v20, other small RNAs and exons. The remaining miRNA results were filtered by the miRDeep* score, a measure of the likelihood that the miRDeep* result is a true miRNA, where higher scores indicate higher confidence of a miRNA result. In our miRDeep* results, score ranged from -9.98 to as high as 11 million, with a median of -0.85.

The score value to be used as a threshold was determined by estimating false positive rate (FPR) and true positive rate (TPR) using the scores of the 4273 results overlapping with known exons and small RNAs (false positives) and of the 783 results overlapping with known miRNAs (true positives), excluding the 194 results overlapping both annotations. The performance of this method was evaluated with a receiver operating characteristic (ROC) curve, a plot of the FPR vs. TPR as the threshold is varied, shown in Additional file 3. The area under the curve (AUC) of the ROC curve, used as a metric of the quality of the classifier, is 0.82 with this method. A score of 86.13 had an estimated FPR of 0.05 and TPR of 0.45, and was used as the score threshold.

**Fig. 2:**
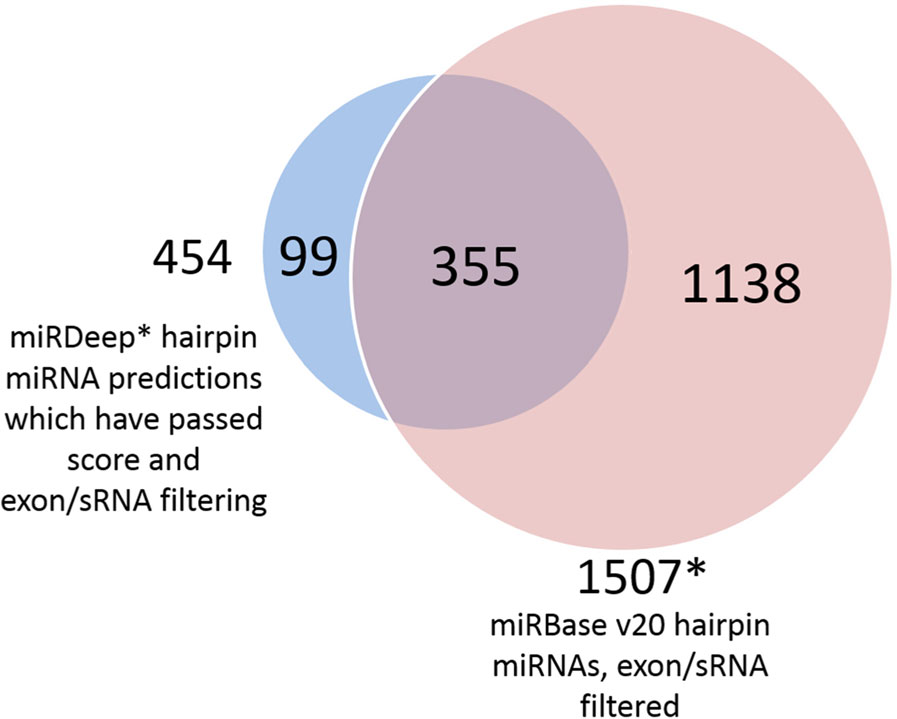
miRDeep* putative miRNAs and miRBase miRNAs. This Venn diagram depicts the overlap between two sets of hairpin miRNAs. The first is the set of miRDeep* results after score-filtering (86.13) and filtering to remove exons and sRNAs, using annotation files from gencode v19 and matching by genomic locations with the intersect function from bedtools v2.22.1. This set of 454 miRDeep* results overlaps the set of miRBase v20 hairpin miRNAs, also filtered for exons and sRNAs with the same method. As seen in the Venn diagram, the majority of the score and exon/sRNA filtered miRDeep* results exist within the miRBase database. The 99 that do not are the final putative novel miRNAs of this study. (*) The total number of filtered miRBase hairpin miRNAs is not the sum of the intersection and the remaining miRBase set because the miRBase and miRDeep* hairpin miRNAs do not have a one-to-one relationship. Twenty-two of the miRDeep* set overlap two members of the miRBase set, and eight of the miRBase set overlap two members of the miRDeep* set. In this Venn diagram we have displayed that 355 of the 454 filtered miRDeep* hairpin miRNA predictions overlap at least one filtered miRBase v20 hairpin miRNA, and 1138 of 1507 filtered miRBase v20 hairpin miRNAs overlap zero filtered miRDeep* hairpin miRNA predictions

**Fig. 3:**
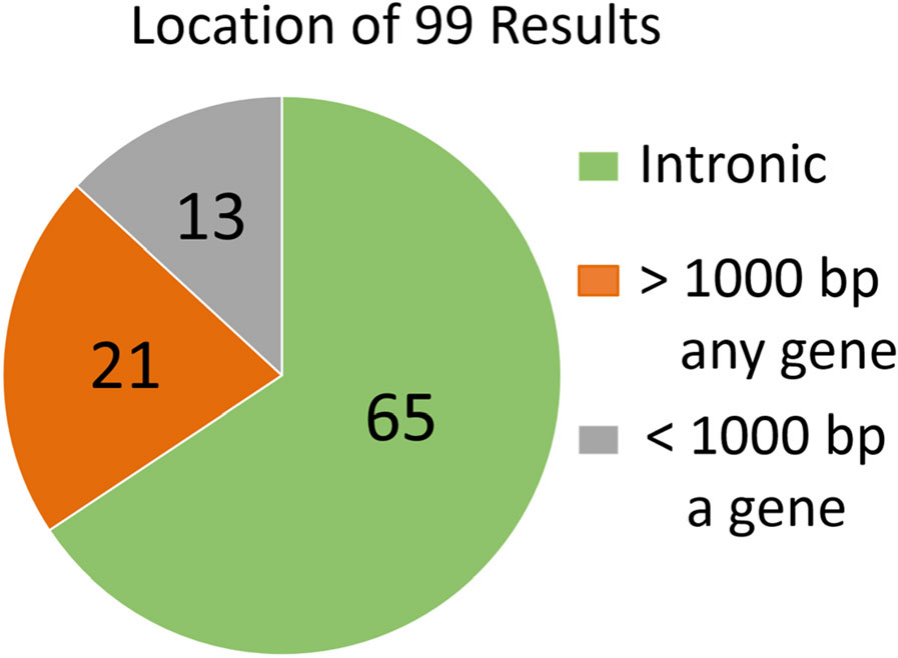
Genomic locations of the putative novel miRNAs. The locations of the 99 novel miRNAs relative to human genes are depicted as a pie chart. It shows the proportion of the 99 novel miRNAs that are within, near (<= 1000 basepairs), or distant (>1000 basepairs) from one or more genes from gencode v19. The 65 novel miRNA results that are within genes are intronic, as cases of miRDeep* results overlapping gencode v19 exon annotations were classified as false positives and excluded

Figure 2 displays a Venn diagram showing the number of score-filtered miRDeep* results which overlap the set of hairpin miRNAs within miRBase v20 (both sets were filtered for exons and other small RNAs). After applying the score threshold of 86.13 to the 3641 novel miRDeep* results, a final list of 99 putative novel miRNAs was identified. Sixty-five of the 99 potential miRNAs were intronic, an additional 13 were within 1000 base pairs of at least one gene, and the remaining 21 were further than 1000 base pairs from any annotated gene from gencode v19. This information is represented as a pie chart in Fig. 3. The 99 results are present within a varying number of the samples, ranging from 2 to all 93, with a mean of 69.4 samples. Sixteen results are present in fewer than half of the samples, while ten are expressed ubiquitously and with a large mean read depth. The distribution of the miRNA results’ mean counts by number of samples present is described in Additional file 4. Raw counts of the novel mature miRNA regions are available in Additional file 5, and summary information on the 99 miRDeep* results is shown in Additional file 6. This includes genomic location, sequence, predicted hairpin secondary structure, miRDeep* score, nearby genes, and summary of differential expression analyses and alignments to miRBase v20 entries.

**Fig. 4:**
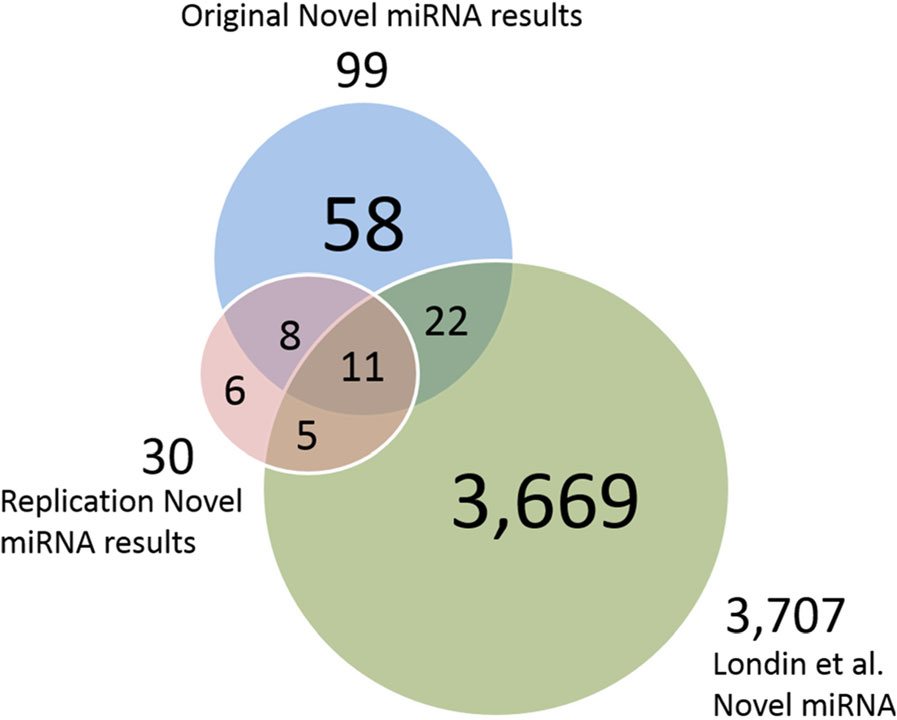
Putative novel miRNAs, replication data miRNAs and Londin et al. miRNAs. This Venn diagram depicts the overlap of three sets of mature miRNAs: the novel mature miRNAs discovered using the HD and PD small RNA sequencing data set, the novel mature miRNAs discovered using the publicly available replication data set, and the Londin et al. mature miRNAs. The intersection of sets was obtained using the intersect function from bedtools v2.22.1 with gene transfer format (gtf) annotations files created by the novel miRNA discovery pipeline and from the supplemental data set of Londin et al. Though the sets vary in size, each has unique miRNA entries and overlap with each other set

Nineteen of the 99 putative novel miRNAs were replicated using two independent human brain small RNA sequencing data sets obtained from the Gene Expression Omnibus (GEO access numbers GSE63501 and GSE46131) [24–26]. Using these publicly available GEO samples (*N* = 36), the replication analysis resulted in 30 putative miRNAs. Matching by overlap of genomic location, 19 of 30 were seen in our original 99 results. The remaining 11 were not found in our data. Thirty-three of the 99 putative novel mature miRNAs were reported in a recent novel miRNA study by Londin et al. [3]. Eleven of the 99 putative miRNAs described in this study appear both in the replication results and in the Londin et al. data set. Figure 4 displays a Venn diagram that depicts the overlap of the 99 novel miRNAs, the 30 replication novel miRNAs and the Londin et al. novel miRNAs data set.

Seven of the 99 putative novel miRNAs plus two known miRNAs that represent the extremes of the expected range of expression were chosen for experimental validation using qRT-PCR (Additional file 7). All 9 were determined to be expressed in an independent set of 12 brains. Each putative novel miRNA was present in at least 7 of the 12 brain samples analyzed. Moreover, the expression levels as determined by qRT-PCR paralleled the relative expression levels determined by small RNA sequencing.

Using the SSEARCH aligner, 34 of the 99 putative novel mature miRNAs align well (e < 0.05 and nucleotide overlap > = 15) to at least one known mature miRNA from miRBase v20. Most of these 34 aligned to 1–4 distinct miRNAs, but 6 aligned to a large number (18–69) of sequences within the miRNA gene family hsa-miR-548. Pairwise alignments of the 99 putative novel mature miRNAs revealed that 34 align to at least one other result. Five of the six sequences that aligned to the hsa-miR-548 family also aligned to each other. Additional file 8 contains information on the alignments to miRBase entries, and Additional file 9 contains information on the pairwise alignments. Two results, chr22_novelMiR_136 and chr22_novelMiR_137, may be the same miRNA. The two have the same mature sequence and location, very similar, large miRDeep* score, but differing hairpin locations. Though they may be distinct transcripts, one was excluded from the differential expression analysis to avoid double-counting the large number of shared reads.

Differential expression analysis of known miRNAs (miRBase v20) plus our 99 putative novel miRNAs was completed using linear models adjusting for age of death. This analysis showed 4 of the 99 putative miRNAs to be differentially expressed between control (*n* = 36) and Huntington’s disease (*n* = 28) samples, and another 3 to be differentially expressed between control (*n* = 36) and Parkinson’s disease (*n* = 29) samples at False Discovery Rate of 5 % (See Table 2). The four HD-associated putative miRNAs have negative log_2_ fold-change values (lower average expression within the disease samples), and of the three PD-associated putative miRNAs, one has a negative log_2_ fold-change and two have positive log_2_ fold-changes.

**Table 2:** Differentially expressed novel miRNAs for Huntington’s disease and Parkinson’s disease

Putative novel miRNA	Contrast	Average log_2_ expression	log_2_FC	*p*-value	FDR-adjusted *p*-value
chr9_novelMiR_203	HD/C	4.03	−1.58	4.91E-04	2.06E-02
chr11_novelMiR_242		2.65	−0.69	2.84E-04	1.40E-02
chr16_novelMiR_272		2.59	−0.76	1.67E-04	1.11E-02
chr18_novelMiR_75		1.32	−0.66	5.83E-04	2.19E-02
chr6_novelMiR_46	PD/C	2.03	0.55	1.58E-03	2.02E-02
chr8_novelMiR_236		1.78	0.53	5.47E-03	4.46E-02
chr9_novelMiR_225		1.92	−0.62	7.20E-04	1.15E-02

The data presented in Table 2 were generated by LIMMA v. 3.22.7

## Discussion

We have discovered 99 putative novel miRNAs, of which 7 have been experimentally validated using qPCR and 19 have been replicated in an independent brain-tissue derived small RNA sequencing data set. Furthermore, 33 of the 99 match novel miRNAs discovered recently by Londin et al. [3], which utilized 1323 samples from 16 tissue types, including 24 from brain tissue. Eleven putative novel miRNAs appear in all three result sets (original, replication and Londin sets), four of which were among the seven experimentally verified. The experimental confirmation of presence within brain samples and the overlap of our findings with those of past studies suggests these novel findings are likely valid miRNAs.

There are several possible reasons why 80 of 99 results were not replicated by the independent data set and 66 of 99 are not seen in the Londin et al. miRNA data set, including differences of disease status, brain region and sample size, and the presence of false positives. The independent data set contains samples from studies of Alzheimer’s disease and dementia but lack HD or PD brains, and one of these studies used temporal neocortex grey matter, while we used prefrontal cortex. The original data set contains 93 brain samples while the replication data set contains 36, and although Londin et al. utilized 1323 small RNA-seq samples, only 24 of these are from brain tissue. Our 93 brain samples are likely to have a greater power to detect potential brain-specific miRNAs, and the Londin et al. data has the capability to detect tissue-specific miRNAs from 15 additional tissues and greater power to detect tissue-nonspecific miRNAs.

Thirty-four of the 99 results aligned well to known miRNAs. These may be novel members of known miRNA families. miRNA family classification is based on sequence (especially seed) similarity, as well as function similarity, conservation and common descent. While we have shown sequence similarity that may indicate potential miRNA familial membership, we cannot definitively determine membership with these data alone. The 65 novel results that did not align well to any currently known miRNA may be novel miRNAs that do not belong to any currently known family.

miRNAs are known to exhibit tissue-specific patterns of expression [3, 8] and because these novel miRNAs have been discovered with pre-frontal cortex samples, they may be specific to the brain or to the prefrontal cortex. Tissue-specificity could explain why these miRNAs have not been previously discovered, as miRNA discovery is not typically performed using brain tissue. However, this may alternatively be explained by our large sample size and the sequencing depth that our concatenation method invokes rather than by tissue-specific expression.

We performed differential expression analysis of the 99 putative novel miRNAs, of which, after adjusting for multiple comparisons with FDR q-value < 0.05, 3 are significantly differentially expressed for PD and 4 are significantly differentially expressed for HD. These seven are not only putative novel miRNAs and potentially specific to the brain, but are potentially associated with neurodegenerative disease. Five of the seven (including all of the HD-associated miRNAs) have lower average expression within the disease samples relative to control samples. Currently, we cannot know whether these miRNAs are involved in the pathogenic mechanisms of disease or are only indirectly associated. Future studies would be required to understand the role of these seven miRNAs in PD or HD pathogenicity.

It should be noted that the batching of samples was done originally for two separate studies, and that different brain banks specialize in different diseases (e.g. the HBTRC is the largest source of Huntington samples). Consequently, these data by necessity are limited by the association between brain bank and batch, and brain bank and disease status. Although we have attempted to mitigate possible biases by implementing identical sample preparation protocols for the studies and correction of batch effects with the Combat batch correction procedure, these differences may have introduced a bias on gene expression levels. This limitation should be appreciated for the differential expression analyses.

We have developed a method utilizing miRDeep* to discover novel miRNAs from concatenated small RNA sequencing samples. Many existing miRNA discovery tools use samples individually, while others pool samples, similar to our approach [27]. The main advantage to pooling data is that it allows the discovery of lowly expressed miRNAs that have too few reads to be successfully evaluated on a per sample basis [27]. As a consequence of this, the miRNA discovery does not account for the distribution of miRNAs across samples, so that a miRNA may be discovered whether it is expressed ubiquitously or within a subset of samples. A disadvantage to our method is that it will not discover the rare cases of legitimate miRNAs coded within exons, which comprise approximately 5 % of currently annotated miRNAs [3], due to our focus on generating high-confidence predictions.

## Conclusion

We have developed a pipeline that utilizes miRDeep*, pooled RNA sequencing samples and a score filtering method to discover novel miRNAs with high confidence. This pipeline was applied to 93 post-mortem human prefrontal cortex samples, yielding 99 putative novel miRNAs. Seven of these putative novel miRNAs were validated experimentally and 19 were replicated using an independent post-mortem human brain RNA sequencing data set. A subset of the 99 results may be specific to the brain or to the prefrontal cortex, and 7 show differential expression between HD and control or PD and control samples. More than a third of the putative miRNA sequences align well to known miRNAs and may be novel members of known miRNA families, while the remaining may be members of novel miRNA families.

Our discovery and characterization of putative novel miRNAs may add to the rapidly-growing repository of known human miRNAs. These results reinforce the possibility that many miRNAs display tissue-specific expression and will only be discovered once many more tissue types are characterized for miRNA discovery. In addition, miRNAs have been implicated in neurodegenerative diseases, and seven of the miRNAs that we have discovered may not only be specific to the brain, but are associated with neurodegenerative disease in the samples available for the current study. We know very little about these miRNAs, and while they may be only peripherally or coincidentally associated with HD and PD, these results may eventually contribute to the understanding of the molecular mechanisms of these diseases.

## Additional files

Additional file 1: Provides information on each of the 93 samples used in this study. This information includes ID, type (Control/HD/PD), PMI (Post-mortem-interval), age at death, sex, batch, brain bank, and cause of death. (XLSX 13 kb)
Additional file 2: Contains quality control information including the average read quality before and after quality and length filtering, the number of reads used in miRNA discovery and in differential expression analyses, and the number of reads removed for each length and quality filtering step. Length filtering used 15 and 27 nucleotides as threshold for the differential expression analysis and a more strict 18 and 23 nucleotides for the miRDeep* miRNA discovery. The numbers of reads filtered are included for all four thresholds. (XLSX 22 kb)
Additional file 3: Shows the receiver operating characteristic curve from the novel miRNA discovery method. (PNG 29 kb)
Additional file 4: A scatter plot depicting for each putative novel miRNA the number of samples that have at least one read count, vs. the mean count value of those samples. (PNG 15 kb)
Additional file 5: A 99 × 93 table with raw read counts of the 99 putative novel miRNA results, for each of the 93 samples. (XLSX 45 kb)
Additional file 6: Contains summary information of each of the 99 novel putative miRNAs and the 30 miRNAs results from public data, in separate tabs. The following information is included in this file: mature and hairpin sequence loci, miRDeep* score, expression, the number of good-quality alignments to miRBase entries, the name of the miRBase entry with the best alignment, the name and distance (basepairs) of any gene within 1000 basepairs of the novel miRNA, adjusted *p*-value and log_2_ fold-change of the differential expression analyses (C v. HD, C v. PD), whether the novel miRNA overlaps an entry within the Londin et al. novel miRNA dataset, which entries of the HD/PD data set and the public data set results match by location, which novel miRNAs were experimentally validated, mature and hairpin sequence, and predicted hairpin secondary structure. (XLSX 35 kb)
Additional file 7: A table containing information on the qRT-PCR performed with seven novel miRNAs and two known miRNAs. Per miRNA, this information includes mean CT, range of CT, cDNA dilution, the number of samples (of 12) with CT < 40, the average read depth, and primer used. (XLSX 8 kb)
Additional file 8: Contains alignment information from good-quality mature sequence alignments of the 99 putative novel miRNAs to miRBase mature sequence entries. (XLSX 46 kb)
Additional file 9: Contains alignment information from good-quality alignments of pairwise alignments of mature sequences of the 99 putative novel miRNAs. (XLSX 15 kb)

## Abbreviations

BA9: Brodmann Area 9
HD: Huntington’s disease
miRNA: microRNA
PD: Parkinson’s disease
