# Supplementary figures and images for: Novel microRNA discovery using small RNA sequencing in post-mortem human brain
Source: BMC Genomics. 2016 Oct 4;17:776. doi: 10.1186/s12864-016-3114-3 (PMC5050850; doi:10.1186/s12864-016-3114-3)

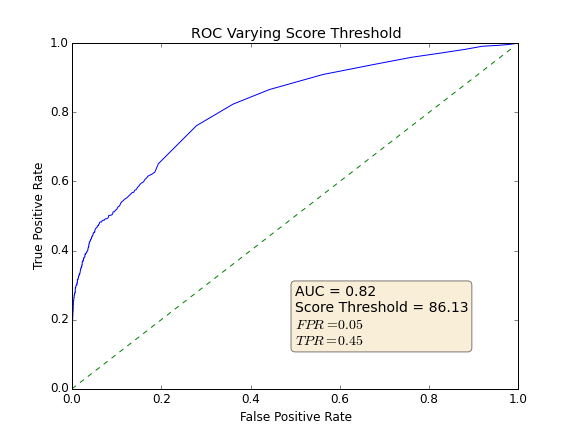

Supplement: Additional file 3: — Shows the receiver operating characteristic curve from the novel miRNA discovery method. (PNG 29 kb) [file 12864_2016_3114_MOESM3_ESM.png]

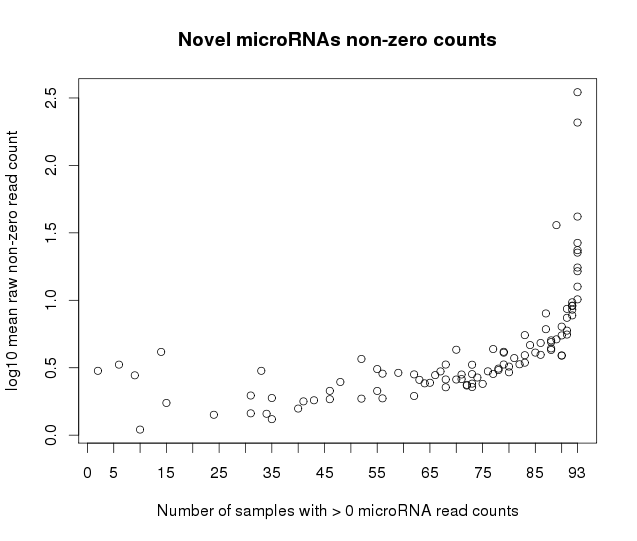

Supplement: Additional file 4: — A scatter plot depicting for each putative novel miRNA the number of samples that have at least one read count, vs. the mean count value of those samples. (PNG 15 kb) [file 12864_2016_3114_MOESM4_ESM.png]
